# Risk Factors and Prognostic Follow-Up of Vasovagal Syncope Children With Seizure-Like Activities During Head-Up Tilt Test Induced-Syncope

**DOI:** 10.3389/fcvm.2022.916542

**Published:** 2022-06-10

**Authors:** Runmei Zou, Shuo Wang, Wen Wen, Hong Cai, Yuwen Wang, Ping Liu, Fang Li, Ping Lin, Cheng Wang

**Affiliations:** ^1^Department of Pediatric Cardiovasology, Children's Medical Center, The Second Xiangya Hospital, Central South University, Changsha, China; ^2^Department of Neonatology, Xiangya Hospital, Central South University, Changsha, China; ^3^Department of Pediatrics, The Third People's Hospital of Shenzhen, Shenzhen, China

**Keywords:** follow-up, head-up tilt test, seizure-like activities, vasovagal syncope, children, risk factor

## Abstract

**Objectives:**

To analyze the risk factors associated with seizure-like activities during head up tilt test (HUTT)-induced syncope in children with vasovagal syncope (VVS) and assess the prognosis of these patients.

**Methods:**

This is a retrospective study. VVS children with or without seizure-like activities during HUTT-induced syncope were included in convulsive or non-convulsive group. The clinical characteristics, hemodynamic parameters during HUTT-induced syncope and follow-up data were reviewed from the HUTT case report form and analyzed.

**Results:**

68 cases (25 males, mean age 11.86 ± 3.35 years) were enrolled in convulsive group and 65 cases in non-convulsive group (24 males, mean age 11.64 ± 2.11 years). There were statistical differences in history duration, response type, and asystole between the two groups (all *P* < 0.05). Fully adjusted logistic regression showed that the risk of seizure-like activities was increased by 37.18 folds for patients with asystole compared with those without asystole (*P* = 0.005), by 308.25 and 6.08 folds for patients with cardioinhibitory type or mixed type compared with vasoinhibitory type (*P* < 0.01). No significant difference was exhibited in negative HUTT conversion rate and the proportion of re-syncope patients between the two groups at follow-up (both *P* > 0.05). None of these convulsive patients underwent pacemaker implantation during follow-up.

**Conclusions:**

Asystole and response type were independent risk factors associated with seizure-like activities. Patients with asystole and mixed or cardioinhibitory responses to HUTT should be closely concerned. However, VVS children with seizure-like activities did not have a poor prognosis at follow-up.

## Introduction

Vasovagal syncope (VVS) is the most common cause of neurally mediated syncope in children and adolescents, which presents with an inability to maintain postural tone and a very brief loss of consciousness, followed by spontaneous recovery (1, 2). VVS is characterized by excessive vagal tone and sympathetic withdrawal, and usually triggered by prolonged standing, emotional stress, postural change and pain, and a crowded environment (3). Head-up tilt test (HUTT) is an important tool to assess autonomic function and applied to differential diagnosis of neurally mediated syncope in pediatric patients (4). A diagnosis of VVS is based on the typical manifestations and a positive response to HUTT that the patient has an episode of syncope or pre-syncope accompanied with hypotension or bradycardia, or both during HUTT (3). Patients with VVS may present myoclonic jerky movements or upward deviation of eyeball during syncopal episodes and be misdiagnosed as epilepsy (5, 6). Besides, reports showed that 8 to 66% of patients with neurally mediated syncope experienced seizure-like activities at the time of syncope during HUTT (7, 8). The hemodynamic parameters related to seizure-like activities during HUTT-induced syncope in adults were reported in the previous study (7). However, there is no relevant study in children and the prognosis of VVS children with seizure-like activities during HUTT-induced syncope has not been revealed.

In this study, we investigated risk factors associated with seizure-like activities during HUTT-induced syncope in VVS children. Furthermore, we evaluated the prognosis of these patients through a follow-up.

## Study Population and Methods

### Study Population and Data Collection

**Figure 1:**
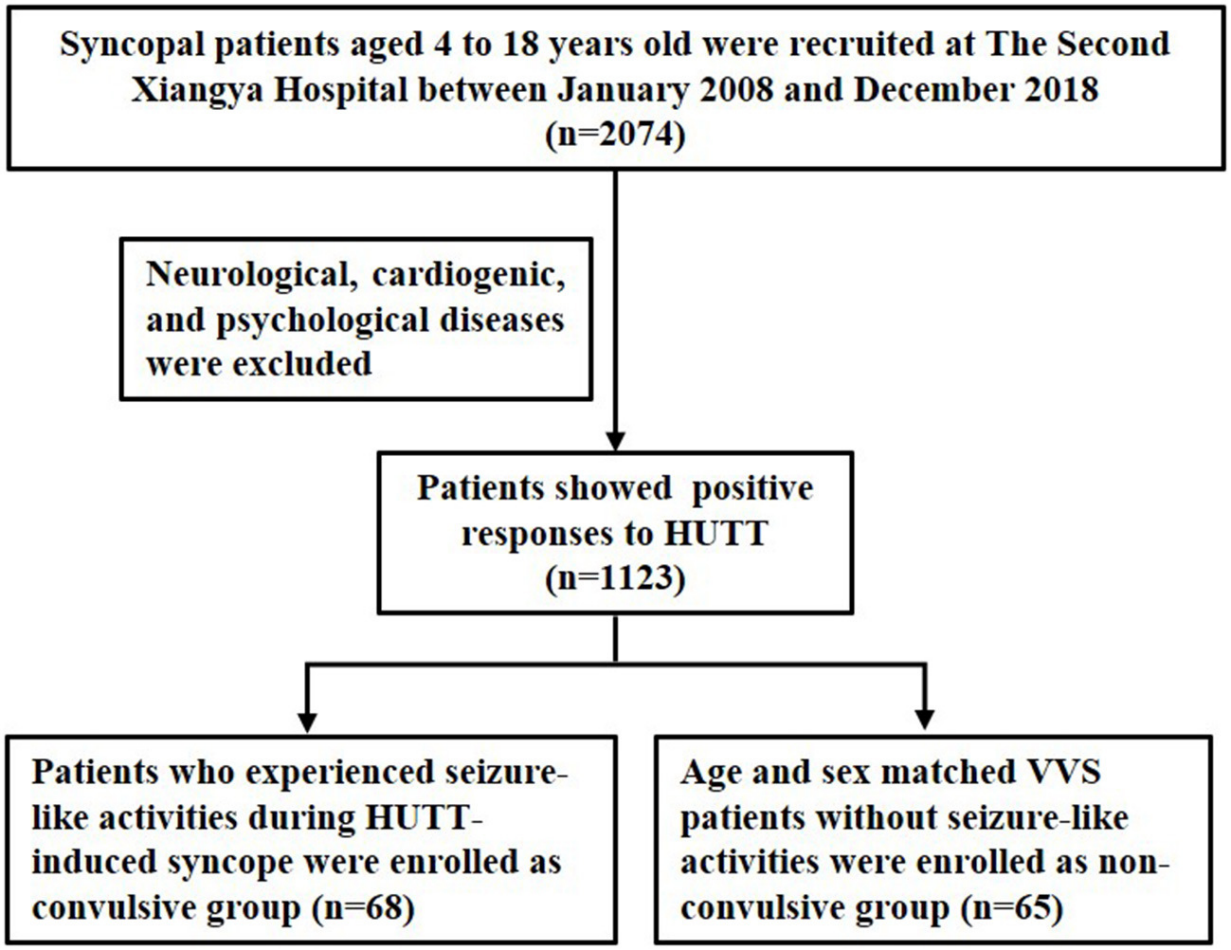
Flow chart of the study population inclusion.

Syncopal patients who presented positive responses to HUTT and diagnosed with VVS were recruited at The Second Xiangya Hospital, Central South University between January 2008 and December 2018. Medical records and HUTT data were retrospectively reviewed by two researchers in our clinic who were blinded to the study protocol. Patients with neurological, cardiogenic, and psychological diseases were excluded. VVS patients who experienced seizure-like activities during HUTT-induced syncope were enrolled as convulsive group. Age and sex matched VVS patients without seizure-like activities during HUTT process were enrolled as non-convulsive group. The inclusion process was shown as Figure 1.

### HUTT Protocol

The protocol of HUTT was performed according to the previous study (9). The test was approved by the Ethics Committee of the Second Xiangya Hospital, Central South University [Ethical Audit No. Study 012 (2014)]. Informed consent was signed by all the subjects or by their guardians prior to the test. Subjects lay still for 10 min, and then basic heart rate (HR), blood pressure (BP), and electrocardiogram (ECG) were recorded. Subjects were tilted at 60° head upward. HR, BP, and ECG were recorded continuously until either 45 min duration or development of syncope or intolerable near syncope symptoms. If syncope occurred, subjects were rapidly put in the supine position. If syncope or presyncope did not occur, tilted posture was maintained, subjects were sublingually medicated with nitroglycerin (4–6 μg/kg, maximum dosage ≤ 300 μg), and HR, BP, and ECG were recorded until for 20 min or syncope or presyncope occurred.

VVS was defined as the development of syncope or presyncope accompanied by any of the following responses (9): (1) hypotension: systolic BP <80 mmHg, and/or diastolic BP <50 mmHg, or over 25% decrease in mean BP. (2) bradycardia: HR <75 bpm for children aged 4–6 years, <65 bpm for those aged 6**–**8 years, and <60 bpm for those older than 8 years. (3) ECG showed sinus arrest, premature junctional contractions. (4) atrioventricular block and asystole ≥ 3 s. There are three response types: vasoinhibitory type, cardioinhibitory type and mixed type. Vasoinhibitory type was characterized by a significant decrease in BP without obvious HR reduction, cardioinhibitory type by a significant decrease in HR without marked BP decrease, and mixed type by both a HR and BP decrease.

### Seizure-Like Activities During HUTT-Induced Syncope

HUTT case report form was reviewed to collect the information on seizure-like activities during HUTT-induced syncope. Seizure-like activities were described by pediatric attending physician. Seizure-like activities were defined as myoclonic jerky movements or tonic-clonic activity of limbs with or without upward deviation of eyeball during HUTT-induced syncope. Myoclonic jerky movements were abrupt, transient, and shock-like muscle movements and classified as focal myoclonic jerky movements or multifocal myoclonic jerky movements (10). When patients experienced seizure-like activities, they were rapidly placed in the supine position. Strategies including keeping the airways open and oxygen therapy infusion were performed. Atropine was utilized to treat asystole when necessary.

### Treatment and Follow-Up

All these VVS patients received health education (avoidance of triggers, early recognition of prodromal symptoms, increased intake of water and salt and careful avoidance of agents that lower BP including diuretic), tilt training (standing against a wall with the united ankles 15 cm from the wall, twice a day) and oral rehydration salts (ORS) for at least 1 month (11, 12). At follow-up, whether the patients experienced re-syncope or re-convulsion during HUTT, and the information on the responses to HUTT were collected from the HUTT case report form.

### Statistical Analysis

Continuous variables for data following normal distribution were described as mean ± SD and analyzed by Student *t*-tests. Continuous variables for data not following normal distribution were expressed as the median with interquartile range and analyzed using the Mann-Whitney U test. Categorical data were described by frequencies and percentages, and analyzed using *x*^2^ test or Fisher's exact test. Multiple logistic regression was utilized to analyze the association between asystole, response type and seizure-like activities. Two models were constructed to illustrate the stability of the relation: Model I in asystole adjusted for sex, age, height and weight; Model II in asystole adjusted for sex, age, height, weight, response type, basic HR, basic systolic BP, basic diastolic BP, time of positive response, recovery time, history duration and frequency of syncope. Model I in response type adjusted for sex, age, height and weight; Model II in response type adjusted for sex, age, height, weight, asystole, basic HR, basic systolic BP, basic diastolic BP, time of positive response, recovery time, history duration and frequency of syncope. Kaplan-Meier curve was used to analyze the difference in ratio of re-syncope patients between non-convulsive and convulsive patients during follow-up. All the analyses were performed with the statistical software packages R (version 3.6.1) (http://www.R-project.org, The R Foundation) and EmpowerStats (http://www.empowerstats.com, X&Y Solutions, Inc, Boston, MA). *P*-value <0.05 was considered to be a statistically significant difference.

## Results

68 VVS patients [mean age 11.86 ± 3.35 years, 25 males (36.77%)] with seizure-like activities during HUTT-induced syncope were included in convulsive group. 65 VVS patients [mean age 11.64 ± 2.11 years, 24 males (36.92%)] without seizure-like activities during HUTT were included in non-convulsive group. The clinical characteristics of VVS patients with or without seizure-like activities were presented in Table 1. There was no significant difference in age, sex, height, weight, frequency of syncope, basic systolic BP, basic diastolic BP, basic HR, time of positive response, recovery time, systolic BP of syncope and diastolic BP of syncope between the two groups (all *P* > 0.05). There were statistical differences in history duration, response type and asystole between the two groups (all *P* < 0.05). HR of syncope in convulsive group was obviously lower than that of non-convulsive group (49.14 ± 21.04 bpm vs. 80.57 ± 29.29 bpm, *P* < 0.001). The ratio of mixed or cardioinhibitory type was higher in convulsive group than that of non-convulsive group (61.77 vs. 35.39%; 27.94 vs. 3.08%, both *P* < 0.001). Convulsive patients had a higher proportion of asystole than non-convulsive patients (39.71 vs.1.54%, *P* < 0.001).

**Table 1:** The clinical characteristics of VVS patients with or without seizure-like activities [Mean ± SD, M (Q1–Q3), *n* (%)].

	**Non-convulsive group (*n* = 65)**	**Convulsive group (*n* = 68)**	***P*-value**
**General information**			
**Sex**			0.985
Male	24 (36.92%)	25 (36.77%)	
Female	41 (63.08%)	43 (63.24%)	
Age (years)	11.64 ± 2.11	11.86 ± 3.35	0.650
Height (cm)	150.17 ± 13.09	149.76 ± 16.54	0.875
Weight (kg)	39.50 ± 11.43	40.25 ± 12.57	0.721
History duration (month)	6.00 (1.00–19.50)	12.00 (6.00–36.00)	0.022
Frequency of syncope (times)	2.00 (1.00–4.00)	3.00 (2.00–4.00)	0.260
**HUTT data**
Basic HR (bpm)	77.66± 12.65	77.46 ± 15.77	0.934
Basic SBP (mmHg)	106.75 ± 10.54	108.07 ± 10.94	0.480
Basic DBP (mmHg)	66.82 ± 7.78	67.02 ± 5.98	0.868
Time of positive response (min)	48.50 (39.75-51.00)	47.00 (25.50-49.00)	0.186
Recovery time (min)	2.00 (1.00-2.00)	2.00 (1.00-2.25)	0.314
HR of syncope (bpm)	80.57 ± 29.29	49.14 ± 21.04	<0.001
SBP of syncope (mmHg)	66.23 ± 25.91	66.68 ± 20.44	0.710
DBP of syncope (mmHg)	36.75 ± 15.35	37.86 ± 18.79	0.790
**Response type**			<0.001
Vasoinhibitory type	40 (61.54%)	7 (10.29%)	
Cardioinhibitory type	2 (3.08%)	19 (27.94%)	
Mixed type	23 (35.39%)	42 (61.77%)	
**Asystole**			<0.001
No	64 (98.46%)	41 (60.29%)	
Yes	1 (1.54%)	27 (39.71%)	

*HUTT, head up tilt test; SBP, systolic blood pressure; DBP, diastolic blood pressure; HR, heart rate*.

The univariate analysis for seizure-like activities was shown in Table 2. We found a positive association between history duration and seizure-like activities, and with 1 month increase in history duration, the risk of seizure-like activities was increased by 2% (*P* = 0.027). The risk of seizure-like activities was higher for patients with cardioinhibitory type or mixed type compared with vasoinhibitory type (increased by 53.29 and 9.44 folds, respectively, both *P* < 0.001). The risk of seizure-like activities was higher for patients with asystole compared with those without asystole (increased by 41.15 folds, *P* < 0.001).

**Table 2:** Univariate analysis for seizure-like activities [*n* = 133, Mean ± SD, M (Q1–Q3), *n* (%)].

	**Statistics**	**OR(95%CI)**	***P*-value**
**General information**
**Sex**			
Male	49 (36.84%)	1.0	
Female	84 (63.16%)	1.01 (0.50, 2.04)	0.985
Age (years)	11.76 ± 2.80	1.03 (0.91, 1.16)	0.648
Height (cm)	149.96 ± 14.89	0.99 (0.98, 1.02)	0.873
Weight (kg)	39.89 ± 11.99	1.01 (0.978, 1.04)	0.718
History duration (month)	10.00 (2.00-36.00)	1.02 (1.00, 1.04)	0.027
Frequency of syncope (times)	2.00 (1.00-4.00)	0.94 (0.85, 1.05)	0.270
**HUTT data**
Basic HR (bpm)	77.56 ± 14.28	0.99 (0.98, 1.02)	0.934
Basic SBP (mmHg)	107.43 ± 10.73	1.01 (0.98, 1.05)	0.477
Basic DBP (mmHg)	66.92 ± 6.89	1.01 (0.96, 1.06)	0.867
Time of positive response (min)	48.00 (30.00–49.00)	0.98 (0.96, 1.01)	0.186
Recovery time (min)	2.00 (1.00–2.00)	1.14 (0.88, 1.47)	0.318
**Response type**			
Vasoinhibitory type	47 (35.34%)	1.0	
Cardioinhibitory type	21 (15.79%)	54.29 (10.29, 286.51)	<0.001
Mixed type	65 (48.87%)	10.44 (4.03, 26.99)	<0.001
**Asystole**			
No	105 (78.95%)	1.0	
Yes	28 (21.05%)	42.15 (5.52, 321.89)	<0.001

*HUTT, head up tilt test; SBP, systolic blood pressure; DBP, diastolic blood pressure; HR, heart rate*.

To further investigate the independent effect of asystole and response type on seizure-like activities based on the results of univariate analysis, we constructed two models (Table 3). In the two models, asystole and response type showed a stable correlation with seizure-like activities (compared with univariate analysis). Therefore, in the fully adjusted Model II for asystole, the risk of seizure-like activities for patients with asystole was increased by 37.18 folds compared with those without asystole (*P* = 0.005). In the fully adjusted Model II for response type, the risk of seizure-like activities for patients with cardioinhibitory type or mixed type was increased by 308.25 folds and 6.08 folds compared with vasoinhibitory type (all *P* < 0.01).

**Table 3:** The relationship between asystole, response type and seizure-like activities in different models [OR (95%CI)].

	**Asystole**		**Response**		
	**No**	**Yes**	**Vasoinhibitory type**	**Cardioinhibitory type**	**Mixed type**
Model I	1.0	49.64 (6.157, 400.22)	1.0	77.01 (13.13, 451.77)	10.34 (3.87, 27.62)
*P*-value		<0.001		<0.001	<0.001
Model II	1.0	38.18 (3.05, 477.48)	1.0	309.25 (19.83, 4823.64)	7.08 (1.84, 27.20)
*P*-value		0.005		<0.001	0.004

*Result variable: seizure-like activities*.

*Exposure variable: asystole/response type*.

*Model I in asystole adjusted for sex, age, height, weight*.

*Model II in asystole adjusted for sex, age, height, weight, response type, basic HR, basic SBP, basic DBP, time of positive response, recovery time, history duration, frequency of syncope*.

*Model I in response type adjusted for sex, age, height, weight*.

*Model II in response type adjusted for sex, age, height, weight, asystole, basic HR, basic SBP, basic DBP, time of positive response, recovery time, history duration, frequency of syncope*.

**Figure 2:**
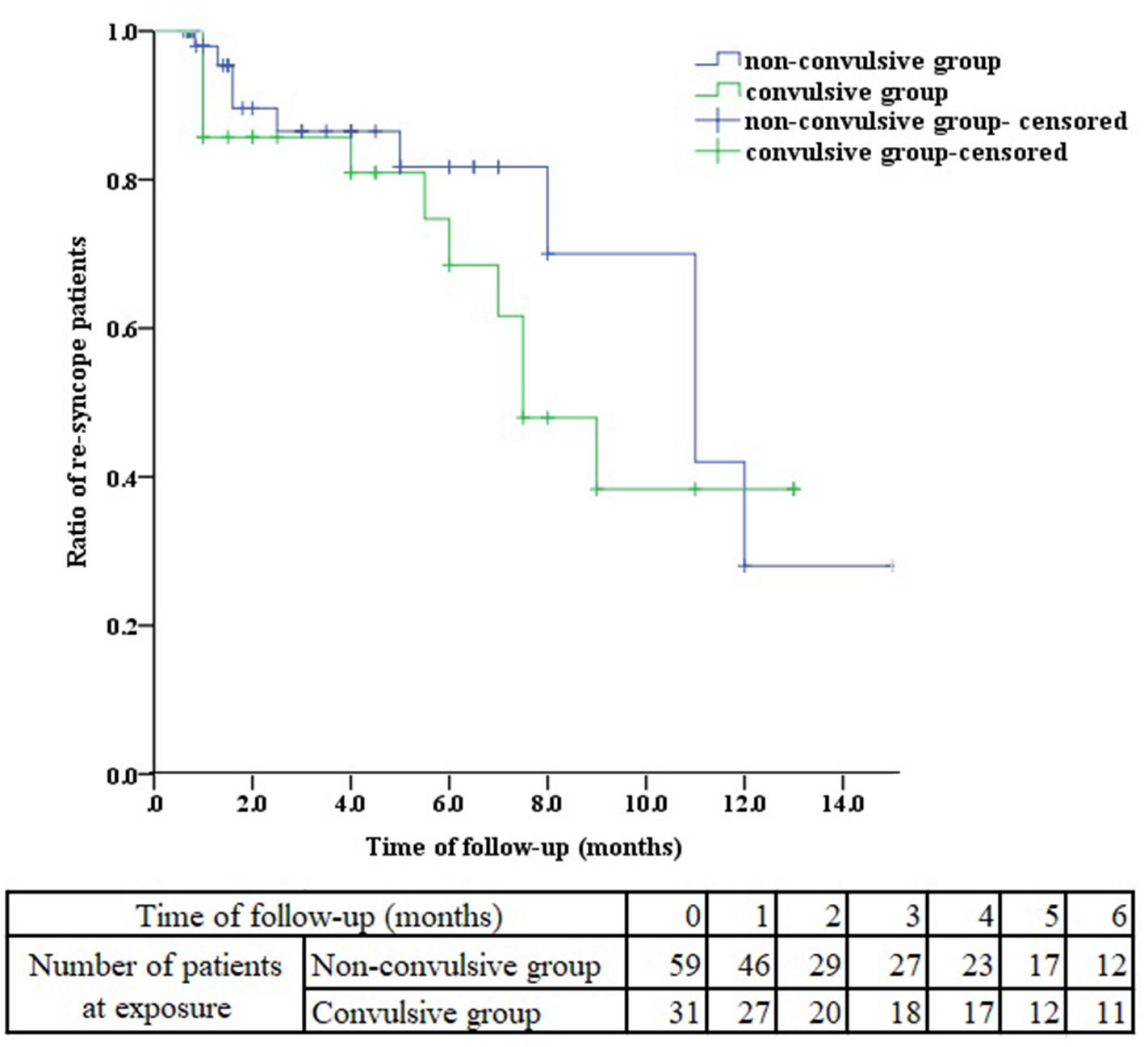
Kaplan-Meier curve analysis of the ratio of re-syncope patients between non-convulsive and convulsive groups.

All these patients received health education, tilt training and ORS for at least 1 month. We performed a follow-up of these VVS patients with or without seizure-like activities. 59 patients in non-convulsive group were followed up and 31 patients in convulsive group. As presented in Table 4, there was no significant difference in follow-up time (median 2.25 months vs. 4.00 months, *P* = 0.226), negative HUTT rate (50.80 vs. 58.06%, *P* = 0.514), the ratio of re-syncope patients (18.64 vs. 32.25%, *P* = 0.147) during HUTT between non-convulsive and convulsive groups. Further, we observed the ratio of re-syncope patients from the Kaplan-Meier curve, there was no difference between the two groups during follow-up (Figure 2, log-rank *P* = 0.364). 9 patients in convulsive group had seizure-like activities during HUTT-induced syncope at follow-up, among which one patient experienced asystole during HUTT-induced syncope. However, none of these convulsive patients underwent pacemaker implantation during follow-up.

**Table 4:** Follow-up of vasovagal syncope with or without seizure-like activities.

**Variables**	**Non-convulsive group**	**Convulsive group**	***P-value***
Follow-up cases	59	31	-
Follow-up time, M(Q1–Q3), month	2.25 (1.00–5.00)	4.00 (1.00–7.50)	0.226
Negative HUTT, *n*	30 (50.80%)	18 (58.06%)	0.514
Re-syncope, *n*	11 (18.64%)	10 (32.25%)	0.147
Re-convulsion, *n*	0	9	-

*HUTT, head-up tilt test*.

## Discussion

In the present study, we found that VVS children with seizure-like activities had a higher proportion of mixed or cardioinhibitory responses to HUTT, and showed a lower heart rate and a higher proportion of asystole at the time of syncope compared with patients without seizure-like activities. Logistic regression analysis suggested that patients with asystole during HUTT-induced syncope had higher risk of seizure-like activities compared with those without asystole. Mixed type or cardioinhibitory type increased the risk of seizure-like activities relative to vasoinhibitory type. However, there was no significant difference in negative HUTT conversion rate and the proportion of re-syncope patients between convulsive and non-convulsive VVS patients during follow-up.

Seizure-like activities, such as eyeball deviation or myoclonic jerky movements were common during HUTT-induced syncope in previous studies. 67% of patients with recurrent unexplained seizure-like activities had positive responses to HUTT and experienced tonic-clonic seizure-like activities during HUTT-induced syncope. HUTT of these patients become negative after treatment (5). The mechanism of seizure-like activities during HUTT-induced syncope in patients with VVS is due to cerebral ischemia, which is secondary to hypotension, bradycardia, or both (13). Cerebral ischemia inhibits telencephalon and other cortical structures, leading to activation of the subcortical structures, especially the brainstem reticular formation, then convulsion develops (14).

Previous studies suggested that seizure-like activities are associated with hemodynamic parameter changes during HUTT-induced syncope. Passman et al. demonstrated that patients with seizure-like activities had significantly lower systolic BP and HR, and more frequent asystole at the time of syncope during HUTT than those without seizure-like activities (8). In another study, patients with seizure-like activities during HUTT had a lower HR and more occurrence of asystole at the time of syncope (7). In our study, VVS children with seizure-like activities showed a lower HR at the time of syncope, but no significant difference in systolic BP was found between patients with or without seizure-like activities. More patients with seizure-like activities had mixed or cardioinhibitory responses to HUTT compared with non-convulsive patients. Besides, the proportion of asystole during HUTT-induced syncope was higher in patients with seizure-like activities. Logistic regression analysis demonstrated that the risk of seizure-like activities for patients with asystole during HUTT was increased compared with those without asystole. Compared with vasoinhibitory type, mixed or cardioinhibitory responses increased the risk of seizure-like activities during HUTT-induced syncope. These results suggested that VVS patients with seizure-like activities had more severe hemodynamic changes at the time of syncope. Asystole and response type were independent risk factors associated with seizure-like activities. Nevertheless, it is unclear whether seizure-like activities are associated with the prognosis of VVS children.

We conducted a follow-up of VVS children with seizure-like activities during HUTT induced-syncope. At follow-up, no significant difference was observed in negative HUTT conversion rate and the proportion of re-syncope patients between convulsive patients and non-convulsive patients. Though 9 patients of convulsive group had seizure-like activities during HUTT-induced syncope, none of these patients underwent pacemaker implantation. VVS is benign and has no effect on mortality. Reports suggested that 17.5% of adult patients presenting an abnormal response suffered a period of asystole during HUTT (15). HUTT induced asystole did not imply a poor outcome but most patients improved spontaneously without pacemaker implantation over a long-term period, even if for those with prolonged asystole more than 15 s (16, 17). Though the incidence of asystole in the pediatric population is lower than that in adults, there were similar conclusions that asystole could be managed without pacemaker implantation in children (18, 19).

VVS children with seizure-like activities did not have a poor prognosis compared to those without seizure-like activities, and convulsive patients with asystole at the time of syncope have a good outcome without pacemaker implantation. However, evidence suggested that patients with syncope had cognitive impairment (20). Whether recurrent syncope with seizure-like activities due to cerebral hypoperfusion has an adverse effect on future brain function should be concerned. We should pay more attention to these patients and give them close observation and effective therapy to reduce episodes of syncope and convulsion at the time of syncope. Furthermore, seizure-like activities at the time of syncope should be correctly recognized and should not be misdiagnosed as epilepsy.

This study has several limitations. First, this was a retrospective study and the sample size was small. The follow-up cases of convulsive group were limited due to loss of some cases. Second, the median follow-up time was 2.25 months in non-convulsive group and 4 months in convulsive group. To further evaluate the prognosis of patients with seizure-like activities, a long-term follow-up should be performed. Third, the study suggested that electroencephalography during syncope shows either a “slow-flat-slow” or a “slow” pattern, which is related to the severity of hypoperfusion (21). Simultaneous electroencephalograms or transcranial Doppler may be necessary to monitor brain waves or cerebral perfusion during the convulsion.

In conclusion, asystole and response type were independent risk factors associated with seizure-like activities. Patients with asystole and mixed or cardioinhibitory responses to HUTT should be closely concerned. However, VVS children with seizure-like activities did not have a poor prognosis at follow-up.

## Data Availability Statement

The data analyzed in this study is subject to the following licenses/restrictions: The original contributions presented in the study are included in the article/supplementary material, further inquiries can be directed to the corresponding author. Requests to access these datasets should be directed to CW, wangcheng2nd@csu.edu.cn.

## Ethics Statement

The studies involving human participants were reviewed and approved by the Ethics Committee of the Second Xiangya Hospital, Central South University. Written informed consent to participate in this study was provided by the participants' legal guardian/next of kin.

## Author Contributions

RZ and CW had primary responsibility for the protocol development, patient enrollment, data collecting preliminary data analysis, and writing the manuscript. SW and WW assisted with data analysis, critical revision for important content, and edited the draft. HC, YW, PLiu, FL, and PLin completed the head-up tilt test. CW supervised the design and execution of the study, checked the data analysis, and contributed to a final approval of the manuscript submitted. All authors have read and approved the final manuscript and assumed full responsibility for its contents.

## Funding

This work is supported by grants from Hunan Province Clinical Medical Technology Innovation Guidance Project (2020SK53405, 2020SK53406) and Health and Family Planning Commission of Hunan Province in China [20201217].

## Conflict of Interest

The authors declare that the research was conducted in the absence of any commercial or financial relationships that could be construed as a potential conflict of interest.

## Publisher's Note

All claims expressed in this article are solely those of the authors and do not necessarily represent those of their affiliated organizations, or those of the publisher, the editors and the reviewers. Any product that may be evaluated in this article, or claim that may be made by its manufacturer, is not guaranteed or endorsed by the publisher.
